# Lack of Decline in Childhood Malaria, Malawi, 2001–2010

**DOI:** 10.3201/eid1802.111008

**Published:** 2012-02

**Authors:** Arantxa Roca-Feltrer, Collins J. Kwizombe, Miguel A. Sanjoaquin, Sanie S.S. Sesay, Brian Faragher, Jim Harrison, Karen Geukers, Storn Kabuluzi, Don P. Mathanga, Elizabeth Molyneux, Maganizo Chagomera, Terrie Taylor, Malcolm Molyneux, Robert S. Heyderman

**Affiliations:** Malawi–Liverpool–Wellcome Trust Clinical Research Program, Blantyre, Malawi (A. Roca-Feltrer, C.J. Kwizombe, M.A. Sanjoaquin, S.S.S. Sesay, M. Molyneux, R.S Heyderman);; Liverpool School of Tropical Medicine, Liverpool, UK (A. Roca-Feltrer, M.A. Sanjoaquin, B. Faragher, R.S Heyderman);; Beit CURE International Hospital, Blantyre (J. Harrison);; McGill University, Montreal, Quebec, Canada (K. Geukers);; Ministry of Health, Lilongwe, Malawi (S. Kabuluzi);; University of Malawi College of Medicine, Blantyre (D.P. Mathanga, E. Molyneux, M. Chagomera, T. Taylor);; Michigan State University, East Lansing, Michigan, USA (T. Taylor);; University of Liverpool, Liverpool (R.S. Heyderman)

**Keywords:** malaria, parasites, trends, hospital, urban, children, Malawi, vector-borne infections

## Abstract

In some areas of Africa, health facility data have indicated declines in malaria that might have resulted from increasingly effective control programs. Most such reports have been from countries where malaria transmission is highly seasonal or of modest intensity. In Malawi, perennial malaria transmission is intense, and malaria control measures have been scaled up during the past decade. We examined health facility data for children seen as outpatients and parasitemia-positive children hospitalized with cerebral malaria in a large national hospital. The proportion of *Plasmodium falciparum*–positive slides among febrile children at the hospital declined early in the decade, but no further reductions were observed after 2005. The number of admissions for cerebral malaria did not differ significantly by year. Continued surveillance for malaria is needed to evaluate the effects of the increased malaria control efforts.

## Abstract 2

Despite increased control activities, malaria did not substantially decline.

Malaria is a leading cause of illness and death among children in countries in which it is endemic (*1*). An increasing number of countries in sub-Saharan Africa are rapidly scaling up malaria control interventions as broad programmatic measures designed to achieve Millennium Development Goal 4 (*2*). To reduce malaria effectively, countries should reach at least 70% coverage of the 4 main malaria control tools: long-lasting insecticide-treated bed nets, indoor residual spraying, intermittent presumptive treatment for pregnant women, and prompt treatment with artemisinin-based combination therapy for symptomatic uncomplicated malaria for which parasitemia was confirmed (*3*). As malaria control interventions increase, several reports based on analyses of long-term surveillance data (*4**–**8*) have emerged from countries with long-standing programs that showed a substantial drop in malaria-associated hospitalizations.

Malawi, in south-central Africa, has year-round malaria transmission that peaks during the long rainy season (late November–April) (*9*) and accounts for 30%–40% of all outpatient visits (*2**,**10**,**11*). Since 2005, and with the support of the President’s Malaria Initiative and the Global Fund, Malawi has started to widely scale up malaria control interventions. In 2000, bed net use was 6% nationally; by 2004, a total of 36% of children <5 years of age reportedly had slept under an insecticide-impregnated bed net during the previous night (*12*). Since 2007, ≈3 million extra nets have been distributed free of charge through health facilities, reaching ≈60% coverage (*13*). Coverage of indoor residual spraying, however, remained low in 2010, even in urban areas (e.g., 3% in Blantyre city) where it was limited mainly to the private sector (*13*). Artemisinin-based combination therapies were adopted as the recommended method of treatment in November 2007. Despite increasing efforts, according to the most recent World Malaria Report, no evidence exists of decreased malaria since 2000 in Malawi (*14*).

Even with the implementation of an improved Health Management Information System in 2002, obtaining complete facility-based routine data remains a challenge in Malawi (*15*). Time trends in health facility records may show evidence of fewer malaria cases and may provide a useful complementary approach to monitor disease changes in settings where routine surveillance systems are incomplete.

Malaria has been studied extensively since the late 1990s at the Queen Elizabeth Central Hospital (QECH) in Blantyre, Malawi (*16**–**19*). In 2001, an improved Pediatric Accident and Emergency Unit (PAEU) was opened, and routine malaria testing of all febrile children in the unit was introduced. A high-dependency research ward in the Department of Pediatrics has been fully functional since 1987 during January–June each year, with the main aim of improving care and undertaking research on severe malaria during the peak malaria season. This arrangement allows monitoring of malaria at a health facility level. We report trends in outpatient visits by malaria parasite–positive children and in admissions for cerebral malaria among children during the past 10 years in the main referral public hospital of Blantyre, Malawi.

## Methods

### Study Site and Population

We examined health facility data for children in the QECH PAEU and for children admitted with cerebral malaria to the high-dependency research ward at QECH during January 2001–December 2010. To provide information about background rates of asymptomatic parasitemia in nonfebrile children, we monitored trends in the prevalence of parasitemia among children admitted for elective surgery to Beit CURE International Hospital (BCIH), an orthopedic hospital in Blantyre, where all patients are routinely tested at admission for parasites.

QECH is a 1,250-bed, government-funded central hospital that provides primary to tertiary care and admits an average of 50,000 patients per year. QECH serves a population of ≈1 million in Blantyre, surrounding townships, and outlying villages. Most hospitalizations are for community-acquired illnesses, and patients admitted are largely either self-referred or referred from a primary health center within Blantyre. Malaria diagnosis in both the PAEU and research ward was performed by the same personnel, who received regular refresher training throughout the study period. BCIH is a 66-bed teaching hospital that specializes in treating the orthopedic needs of children and adults. It is a referral hospital and therefore covers a population from both urban and rural areas in the same region. Health care is provided free to children at both facilities.

### Data Abstraction

From every child in the PAEU who had a febrile illness, blood was obtained for a thick blood film malaria parasite examination and for measurement of hematocrit. These criteria did not change during the study period. Total monthly numbers of outpatient visits to QECH by children, malaria slides taken, and results of thick-film microscopy were available from the PAEU laboratory records. To obtain estimates of parasite density and blood hematocrit levels, we abstracted individual-level records from January 2001 through December 2010. Because of the large volume of data recorded since 2001, we abstracted data from only 2 days (Monday and Thursday) of the first and last weeks of each month.

The criteria for admission into the research ward with cerebral malaria were consistent with World Health Organization guidelines (*20*) and remained unchanged over time. Diagnostic requirements for cerebral malaria were coma (Blantyre coma score <3) persisting for at least 4 hours after admission, *Plasmodium falciparum* parasitemia, negative cerebrospinal fluid, and no other cause of coma identified on examination or investigation. These children were a subset of children admitted to QECH and represent virtually all children admitted with cerebral malaria each year during the rainy seasons. All the diagnoses were reviewed daily by an experienced clinician.

Records from BCIH were available only from 2003 onward. The proportion of children admitted to this hospital for elective surgery who had parasitemia served as a proxy measure of the prevalence of parasitemia in the referred community (*5*) because children attending a hospital offering free orthopedic care are unlikely to be biased with regard to malaria risk.

### Statistical Methods

We used Stata version 10.1 (StataCorp LP, College Station, TX, USA) for descriptive and statistical analyses. Monthly slide positivity rates (SPRs) were calculated as the proportion of positive slides of all slides in a given month. Monthly figures for the prevalence of severe anemia (packed cell volume [PCV] <15), moderate-to-severe anemia (PCV 15%–24%), and mild (PCV 24%–33%) anemia also were obtained for the same period. Approximate parasite densities were based on numbers of asexual-stage *P. falciparum* parasites per oil-immersion field on the thick blood film (*21**,**22*): + = 1–10 parasites/100 fields; ++ = 11–100 parasites/100 fields; +++ = 1–10 parasites/field; and ++++ = >10 parasites/field . For our analysis, sick children attending the outpatient clinic who had >1 parasite per field (last 2 categories) were considered to be more likely than others to have a fever that was actually attributable to malaria; changes in the proportion of +/++ and +++/++++ parasite levels over time were therefore assessed by logistic regression. Trends in the malaria SPR and anemia prevalence from the PAEU at QECH, as well as malaria SPR trends from BCIH, were assessed by using logistic regression weighting by the total number of slides taken (for SPRs) or by the total number of children tested (for anemia prevalence). Because of the large number of statistical comparisons performed, differences were considered significant only for p values <0.001, although results with a significance level of 0.05 also were reported. Annual trends of cerebral malaria were tested by Poisson regression for 2001–2010 (dependent variable: cerebral malaria counts; independent variable: 2001–2010, reference year 2001).

## Results

### Slide Positivity Rates among Children in the PAEU

**Figure 1:**
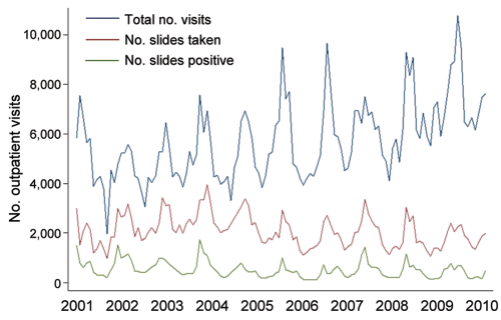
Temporal trends of total monthly outpatient visits, malaria slides taken, and parasitemia-positive slides recorded in the Pediatric Accident and Emergency Unit at Queen Elizabeth Central Hospital, Blantyre, Malawi, 2001–2010.

During January 2001–December 2010, monthly data on the total numbers of outpatient visits, malaria slides, and positive slides were available from the PAEU. During the decade, 686,118 outpatient visits were recorded. Of these, a malaria slide was obtained at 242,953 (35.4%) visits, and 61,320 (25.2%) of these were parasite positive for *P. falciparum*. The total number of outpatient visits in successive years increased gradually, but numbers of slides taken and numbers of positive slides remained approximately constant over time, except for an increase during 2001–2003 (Figure 1). The patterns of outpatient visits for malaria were highly seasonal, with peaks of total positive slides after the main rainy season (December–May) (Figure 1). SPRs peaked in 2001 and 2002 and fell significantly by approximately one third in 2003 and 2004 from 2001 (p<0.001). In 2005 and 2006, SPRs significantly decreased from 2001 by an additional one quarter (p<0.001), but then returned to the 2003 and 2004 levels in 2007–2010 (p<0.001). Although annual SPRs during 2005–2010 were significantly lower than in 2001, annual SPRs did not decline further within the last 6-year period (2005–2010) (Table).

**Table:** Annual numbers of malaria-related outpatient visits and admissions of children with cerebral malaria at QECH and children hospitalized for elective surgery at BCIH who had a parasitemia-positive slide, Malawi, 2001–2010*

Year	QECH						BCIH	
	Outpatients			Research ward			Trauma patients	
	Malaria slides taken	Parasitemia-positive slides, no. (%)†		Cerebral malaria cases‡	Mean patient age, mo (95% CI)		Malaria slides taken	Parasitemia-positive slides, no. (%)§
2001	21,432	7,392 (34.5)		131	34.2 (30.0–38.4)		NA	NA
2002	27,898	9,251 (33.2)¶		123	42.7 (38.0–47.4)		NA	NA
2003	30,238	7,217 (23.9)#		106	45.0 (39.1–50.9)		320	49 (15.3)
2004	24,675	6,429 (26.1)#		124	43.8 (37.7–49.9)		448	50 (11.2)
2005	26,853	4,591 (17.1)#		NA	NA		551	102 (18.5)
2006	20,850	4,062 (19.5)#		86	38.0 (33.0–43.0)		481	80 (16.6)
2007	23,996	5,969 (24.9)#		105	38.3 (32.8–43.9)		651	97 (14.9)
2008	22,907	5,903 (25.8)#		99	51.8 (46.1–57.5)#		755	98 (13.0)
2009	21,126	5,374 (25.4)#		118	47.8 (42.8–52.8)#		406	74 (18.2)
2010	22,978	5,132 (22.3)#		151	48.8 (44.6–53.0)#		420	43 (10.2)^¥^
Total	242,953	61,320 (25.2)		1,043	43.5 (41.8–45.2)		4,032	593 (14.7)

*QECH, Queen Elizabeth Central Hospital; BCIH, Beit CURE International Hospital; NA, not available. †Reference year 2001. ‡Five records had missing information on year. §Reference year 2003. ¶p<0.05. #p<0.001.

### Parasite Density and Anemia Prevalence among Children in the PAEU

**Figure 2:**
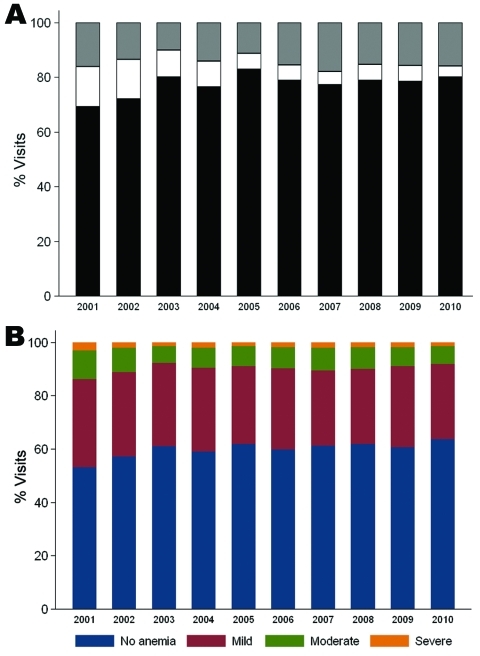
Proportion of parasite density levels (A) and anemia categories (B) over time in the Pediatric Accident and Emergency Unit at Queen Elizabeth Central Hospital, Blantyre, Malawi, 2001–2010.

To analyze parasite densities and hematocrits of children in the PAEU, we abstracted 22,397 records (22,267 with hematocrit information) from the PAEU laboratory books. Books were available for the entire period, except for 2 quarters for which books were missing (October–December 2004 and January–March 2010). No clear differences were observed in parasite density levels over time (Figure 2, panel A). Overall, 15.7% of slides (95% CI 15.2–16.1) had >1 parasite/field (i.e., PAEU visits more likely to be attributable to malaria). This proportion was significantly lower in 2003 (odds ratio [OR] 0.54, 95% CI 0.45–0.64) and 2005 (OR 0.58, 95% CI 0.48–0.69) than in 2001, but no significant differences were seen in the remaining years, and no further reductions were observed after 2005.

The overall prevalences of mild, moderate, and severe anemia were 49.7 (95% CI 49.1–50.4), 12.9 (95% CI 12.5–13.4), and 3.2 (95% CI 2.9–3.4), respectively. Each anemia category peaked in 2001 and significantly fell by 23%, 26%, and 39%, respectively, in 2002 (p<0.001). In 2003, the prevalences of all anemia categories significantly decreased by an additional 13%, 29%, and 23%, respectively (p<0.001). No further reductions occurred after 2004 (Figure 2, panel B).

### Admissions for Cerebral Malaria

During January–June in 2001–2010, a total of 1,048 children with cerebral malaria were admitted to the research ward at QECH; 433 (41%) also had severe malarial anemia (PCV <15%). Data for 2005 were not included because the research ward was not fully functional during that year.

During the 10-year study period, the number of children admitted with cerebral malaria per season ranged from 85 to 150, with the maximum in 2010 (Table). When monthly trends were assessed by Poisson regression and by using 2001 as the reference year, no significant differences were observed by year. The overall linear trend was also tested, but no significant trend was found over time (p = 0.906).

**Figure 3:**
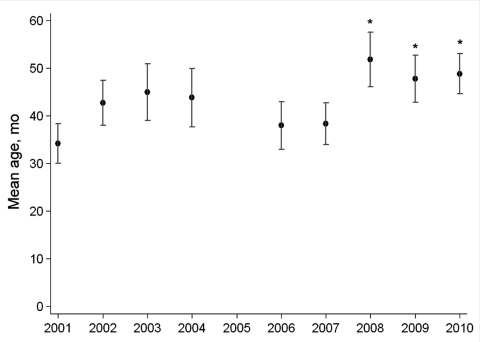
Mean age (95% CI) of children with cerebral malaria admitted to the research ward at Queen Elizabeth Central Hospital, Blantyre, Malawi, during January–June, 2001–2010. Data were not available for 2005. *Denotes a significant difference (p<0.001) in the mean age compared with that in the reference year (2001).

Compared with 2001, the mean age of patients with cerebral malaria did not significantly vary each year up to 2007 but then significantly increased in 2008–2010 (Table; Figure 3). Mean ages during 2008–2010 (49.0 months [95% CI 46.2–51.8 months]) remained significantly higher (p<0.001) than the mean age during 2001–2007 (43.3 months [95% CI 38.2–42.4 months]).

### Malaria Parasitemia in Children Admitted to BCIH

Data from children admitted to BCIH for elective surgery were available for 2003–2010. During this period, a total of 4,033 slides were taken, and an average of 14.7% of patients had parasitemia. Although the SPR varied slightly by year, no significant trend was observed over the 8-year period (Table). Compared with 2003 (reference year), SPRs did not differ significantly for any year except 2010, when SPRs were 27% lower (p<0.05).

## Discussion

In several countries, including Malawi’s neighbor Zambia, health facility data have indicated significantly declining trends in malaria (*4**–**8**,**23**–**25*). Investigators have attributed such declines (at least partially) to the rapid scaling up of malaria control interventions. To our knowledge, this study is one of the few hospital-based retrospective analyses to show no markedly declining trends in malaria during the past decade in a sub-Saharan African country (*26*). The apparently undiminishing malaria at QECH has occurred in the context of increasing efforts to increase malaria control interventions in the country and despite marked reductions in overall deaths among children <5 years of age (*27*). Progress has in fact been substantial in scaling up malaria control interventions in Malawi in recent years, but some parts of the country did not reach high coverage until 2010 (*13*). That evidence of a reduction in malaria in children was not observed in the period during 2001–2010 is therefore not entirely surprising; such a reduction might not become apparent until after 2010. Although use of insecticide-treated bed nets for children <5 years of age has increased quickly in recent years, reaching ≈52% in urban areas and 57% in the southern region (including Blantyre), coverage of other interventions, such as indoor residual spraying, remained low (≈3%) in Blantyre in 2010 (*13*). In areas where dramatic reductions of malaria attributable to rapid scale-up of interventions have been reported (such as in neighboring Zambia), high coverage of interventions with both insecticide-treated bed nets and indoor residual spraying was achieved (*8**,**28**–**30*). Therefore, because indoor residual spraying coverage is likely to play a critical role in reducing transmission intensity and, ultimately, the incidence and prevalence of malaria, in 2011, Malawi expanded its indoor residual spraying activities to 7 districts (*13*) to maximize the effects of malaria control interventions. Strengthened surveillance systems capable of monitoring short-term changes in disease patterns and evaluating in-country differences in uptake of interventions will become key for assessing these expected effects.

The data presented in this retrospective analysis show little change in the pattern of malaria in children during 2001–2010 in Blantyre. The proportion of malaria-positive slides among febrile children in the PAEU declined in 2003 from the preceding 2 years, but no further reductions were observed after 2005. Approximate parasite densities assessed from thick blood films also remained similar throughout the decade. The prevalence of anemia decreased significantly during the first 3 years of the study period, but no further changes of note occurred after 2004. Trends in parasite density and anemia prevalence in children in the PAEU were based on a systematic sample of records. Although a systematic sampling approach could have led to a potential underestimate or overestimation of monthly figures, these potential differences are likely to have been diluted after monthly estimates were pooled to obtain overall annual figures and are therefore unlikely to have distorted true trends of parasite density and anemia levels over time.

The unexpectedly high SPRs and anemia levels in 2001 might have resulted from the severe food crisis that affected the entire country during that period. There were reports at the time of increases in the prevalence of malaria and anemia in the Blantyre area (*31*). Because laboratory records from outpatient visits were not available before that period, we were unable to assess trends before 2001.

The number of children with cerebral malaria admitted each season to QECH has fluctuated moderately since 2001 but with no downward trend and with no significant reduction in any year since 2001. Variations in the rainfall pattern are unlikely to have played a major role in the variations in patients admitted with cerebral malaria. The amount of rainfall was fairly constant from year to year, and the months of peak rain coincided with the months of peak cerebral malaria admissions in all years except 2008 (N. Feasey et al., unpub. data).

The mean age of children with cerebral malaria was significantly higher after 2007 than in 2001. A shift of susceptibility to severe malaria toward older children is theoretically possible in areas where malaria control interventions are being rapidly scaled up and has been reported elsewhere (*5*). Continued monitoring of our patient population is needed to confirm whether this trend is maintained. Unlike the situation in other sites, in Blantyre this recent age trend in cerebral malaria patients has not been accompanied by equivalent changes in SPR or anemia prevalence among hospital outpatients.

The use of health facility–based data is subject to several limitations. These limitations include lack of a known community denominator, likely variations in access to or use of the facility, and uncertain quality and standardization of diagnoses. Such data are best interpreted in the context of community-based surveys, which can provide data on the extent of coverage by various interventions and provide indicators of infection and disease prevalences among the population. We used malaria indicators from children admitted for elective surgery to an adjacent pediatric orthopedic hospital (BCIH) as a proxy for indicators likely to prevail in the community surrounding both hospitals. These data showed no substantial reductions in SPR during 2003–2009 but some evidence of a reduction (p<0.05) in 2010 from 2003. The SPR obtained from these trauma patients is a good proxy for the malaria prevalence in the community (*5*) because children attending a free trauma hospital are unlikely to be a biased sample with regard to malaria risk. Although the catchment area of BCIH is wider than that of QECH (because it admits children from the southern and central regions), and therefore could include children exposed to higher or lower intensities of malaria transmission than those of Blantyre district, the catchment area and referral patterns have not changed over time. Individual SPR trends are therefore unlikely to have been biased in this respect. These data, based on a retrospective health-facility analysis that used a significance level of 0.001, suggest that the persisting levels of *P. falciparum* infection among children with fever at the hospital reflect a similar persistence of the infection in the community over the last decade.

Several factors might have led to underestimation of a decline in malaria prevalence, however. The increase in the number of outpatient visits over time probably reflects population growth of the area or changes in health-seeking behavior (from increased activities in health promotion that would have resulted in increased self-reported outpatient visits), and the number of slides taken remained roughly constant over time. This trend could represent a decrease in the proportion of attendees with a malaria-like illness; alternatively, the facility might have reached its capacity to conduct malaria tests, and when that capacity was exceeded, patients were treated for malaria on the basis of a presumptive diagnosis. If the latter occurred, we would have underestimated malaria in the later years of our study period. This potential underestimation is an intrinsic problem of retrospective health facility–based studies that might have resulted in a systematic bias in observed trends. Although a systematic bias could have occurred in the PAEU, we are confident that it did not affect the high-dependency ward because all cerebral malaria patients are admitted to this ward during the rainy season. However, previous studies reporting declining trends of hospital admissions for malaria (*4**,**6**–**8**,**23**–**25*) also were subject to similar limitations and should be interpreted with caution. Lastly, many children seen at QECH who were residents of Blantyre might have acquired their malaria in their journeys to rural areas because use of bed nets by short-term travelers is less common than for those sleeping in their permanent residence (*32*), which partially could have masked a true decline in malaria in Blantyre.

As malaria control efforts increase in Malawi and in similar areas, continued surveillance is needed to describe changes in malaria among a population exposed to lower intensities of malaria transmission. Significant increases in the mean age of outpatient and hospitalized children with positive slides and a rise in the proportion of cerebral malaria among all those admitted for severe malaria are among changes that might occur (*33*). Field-based surveillance and regular malaria indicator surveys are major methods by which to monitor the effectiveness of malaria control. However, to identify changes in disease patterns, mechanisms for the sustainable, prospective collection of surveillance data from health facilities will be increasingly needed to evaluate the effect of malaria control programs.
